# Murder she said – a review on mental health issues in intimate relationship violence

**DOI:** 10.1192/j.eurpsy.2021.1615

**Published:** 2021-08-13

**Authors:** A. Delgado, A. Cortiñas, A. Sousa

**Affiliations:** Psychiatry, Centro Hospitalar Universitário de São João, Porto, Portugal

**Keywords:** domestic violence, mental health, intimate relationship violence

## Abstract

**Introduction:**

Violence in intimate relationships is a prevalent worldwide health problem and it is underreported, underrecognized and underadressed by health care professionals.This problem affects women more commonly than men and occurs in heterosexual and same-sex relationships. Violence can include physical, emotional, sexual and financial abuse, as well as control over contraception or pregnancy and medical care and it tends to be repetitive, with an escalation in frequency and severity over time. Abused patients exhibit chronic physical and emotional symptoms and injuries resulting from physical and sexual violence.

**Objectives:**

We conducted a review on violence in intimate relationships and the impact on mental health of the victims.

**Methods:**

Comprehensive search of literature in the medical databases MEDLINE, PsycINFO, SciELO using the keywords: women, violence, intimate relationship violence, mental health, self injury.

**Results:**

Research has established a relationship between violence and mental health outcomes among women and girls. Violence or inter-personal trauma render women vulnerable to a range of psychiatric symptoms - depression, post-traumatic stress disorder (PTSD), suicide, and substance use are most common. Women reporting bidirectional violence had higher rates of depression and PTSD. When examining differences in rates of psychiatric disorders by the type of violence, it was found that all types of violence were strongly associated with all types of psychiatric disorders. Severity of psychiatric symptoms increased stepwise with increasing severity of violence.

**Conclusions:**

Caring for patients in abusive relationships can be challeging - continuous supportive care improves patient outcomes. Physicians shoul be able to recognize and manage this situations in order prevent its negative outcomes.

